# High-contrast sub-millivolt inelastic X-ray scattering for nano- and mesoscale science

**DOI:** 10.1038/ncomms5219

**Published:** 2014-06-23

**Authors:** Yuri Shvyd’ko, Stanislav Stoupin, Deming Shu, Stephen P. Collins, Kiran Mundboth, John Sutter, Martin Tolkiehn

**Affiliations:** 1 Advanced Photon Source, Argonne National Laboratory, Argonne, Illinois 60439, USA; 2 Diamond Light Source Ltd, Didcot, Oxfordshire OX11 0DE, UK; 3 DESY, Notkestrasse 85, 22607 Hamburg, Germany

## Abstract

Photon and neutron inelastic scattering spectrometers are microscopes for imaging condensed matter dynamics on very small length and time scales. Inelastic X-ray scattering permitted the first quantitative studies of picosecond nanoscale dynamics in disordered systems almost 20 years ago. However, the nature of the liquid-glass transition still remains one of the great unsolved problems in condensed matter physics. It calls for studies at hitherto inaccessible time and length scales, and therefore for substantial improvements in the spectral and momentum resolution of the inelastic X-ray scattering spectrometers along with a major enhancement in spectral contrast. Here we report a conceptually new spectrometer featuring a spectral resolution function with steep, almost Gaussian tails, sub-meV (≃620 μeV) bandwidth and improved momentum resolution. The spectrometer opens up uncharted space on the dynamics landscape. New results are presented on the dynamics of liquid glycerol, in the regime that has become accessible with the novel spectrometer.

## Abstract (web summary)

Inelastic X-ray scattering suffers from not being able to access high-frequency collective excitations in condensed matter on the nano- to mesoscales. Here, the authors report a new spectrometer that allows these regimes to be accessed, and demonstrate its advances by studying an organic glass-forming liquid.

## Graphical abstract

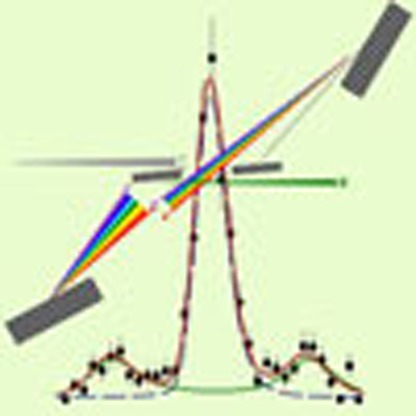

Photon and neutron inelastic scattering probes are indispensable for studies of fast dynamics in condensed matter on atomic to microscopic scales. Despite numerous advances, critical gaps exist in current experimental capabilities. Figure 1 shows that no established probe is able to access collective excitation on the nano- to mesoscale. Entering this uncharted region of dynamics is critical for understanding the nature of glass and the liquid-glass transition, which have an impact on many other fields, including biology, yet remain great unsolved mysteries^1^,^2^,^3^,^4^,^5^,^6^,^7^.

For a long time, since the pioneering experiment of Brockhause and Stewart^8^ in 1958, only inelastic neutron scattering could measure nanoscale collective dynamics in condensed matter. Inelastic neutron scattering permits extremely small energy transfer resolution but rarely permits access to good momentum transfers, and hence it cannot be used to study disordered systems, whose sound velocities are typically 500–7,000 m s^−1^.

The advent of intense synchrotron radiation sources in the 1980s culminated in the first observation of inelastic X-ray scattering (IXS) from phonons^9^. With the third-generation radiation sources of the 1990s, IXS spectrometers were pushed to ≃1.5 meV spectral resolution and ≃1.5 nm^−1^ momentum resolution^10^,^11^, resulted in the first breakthroughs^10^,^12^ and further systematic studies^13^,^14^,^15^,^16^,^17^ of excitations in disordered systems and have since spread worldwide^11^,^18^,^19^,^20^. However, for almost 20 years these resolutions have not improved. The long Lorentzian tails of the spectral resolution function and the low-scattering cross-section severely compromise the ability of the existing spectrometers to detect important low-energy excitations.

Here we report the first implementation of a conceptually new IXS spectrometer, based on new principles of X-ray monochromatization and spectral analysis. We prove the efficacy of combining novel optical components to create an ultra-high-resolution IXS (UHRIX) spectrometer with unmatched performance in terms of energy and momentum resolution, and spectral contrast. The UHRIX spectrometer opens up uncharted space on the dynamics landscape, shown in dark green in Fig. 1. This is precisely the space of vital importance for the science of disordered systems. UHRIX is perfectly optimized for the latest generation of the synchrotron radiation sources and X-ray free electron laser facilities. We have verified the new spectrometer concept by carrying measurements on liquid glycerol at previously inaccessible regions of energy and momentum transfer.

## Results

### Spectrometer optical principles

UHRIX spectrometer is the first implementation of an optical concept, based on new principles of X-ray monochromatization and spectral analysis^21^. It combines flat crystal angular dispersive X-ray optics with collimating and focusing curved mirrors.

**Figure 2:**
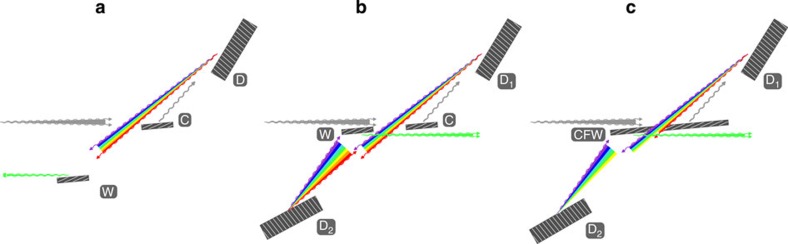
CDW optics: principles and modifications. (**a**) Schematic of the CDW optics, comprising three asymmetrically cut crystals functioning as a collimator—C, a dispersing element—D and a wavelength selector—W, respectively. (**b**) CDDW optics is a modification of the CDW scheme augmented with two dispersing elements *D*_1_ and *D*_2_, ensuring enhanced dispersion rate and in-line scattering geometry. (**c**) CDFDW optics is another modification of the CDW scheme. Crystals C and W are combined into a single CFW crystal executing three key functions: the collimator—C, an anomalous transmission filter—F and a wavelength selector—W, in successive reflections.

The key principle behind UHRIX is to adopt a highly asymmetric Bragg reflection close to backscattering, as an atomic-scale diffraction grating. A relatively large bandwidth is diffracted, but with a large angular dispersion perpendicular to the crystal surface, similar to that in a conventional grating, which diffracts light of different colours into different directions. The strongly divergent ‘rainbow’ of waves from this dispersing element (D) are then intercepted by a Bragg reflection that acts as a wavelength selector (W) by passing only those that emerge within a very narrow angular window—Fig. 2a. The third essential component in this scheme is a collimator (C)—another highly asymmetric Bragg reflection—placed before the dispersing element to ensure that waves incident on the D crystal are close to parallel. The initial collimation-dispersion-{wavelength-selection} (CDW) scheme can be modified to a collimation-dispersion-dispersion-{wavelength-selection} (CDDW) scheme—Fig. 2b—featuring enhanced angular dispersion due to two dispersing elements D_1_, D_2_ and in-line scattering geometry. With D-crystals set into extreme backscattering Bragg diffraction, the CDDW transforms into collimation-dispersion-{anomalous-transmission-filter}-dispersion-{wavelength-selection} (CDFDW) scheme with crystals C and W combined into a single collimation-{anomalous-transmission-filter}-{wavelength-selection} (CFW) crystal playing simultaneously the roles of the collimator (C), the anomalous transmission filter sharpening the tails of the spectral function (F) and wavelength selector (W), in three successive asymmetric Bragg reflections^22^,^23^.

The CDW optics and their modifications provide a very narrow spectral resolution function with steep tails, in combination with unusually large angular acceptance. It performs best at photon energies of ≃5–10 keV, where undulator flux and momentum resolution are very high. The large angular acceptance, ~\n100 μrad, makes possible to combine the CDW optics with large-angular acceptance collimating optics into a large-angular acceptance analyser system.

**Figure 3:**
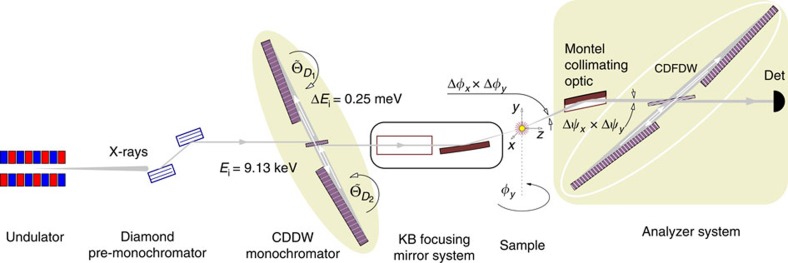
UHRIX layout. Layout of the UHRIX instrument in the vertical (*y*,*z*) scattering plane showing all optical elements and all 15 deflections of the X-ray beam. Rotation of the analyser system in the horizontal plane (*x*,*z*) by an angle *ϕ*_*y*_ about the vertical *y* axis passing through the sample provides access to photons scattered with a momentum transfer *Q*=2*k* sin(*ϕ*_*y*_/2). See Methods for more details.

The complete UHRIX spectrometer (Fig. 3) comprises a CDDW-type monochromator and a CDFDW-type analyser, based on the CDW operating principle. However, the analyser is more challenging as the ‘source’ is a diverging beam from the sample, rather than a parallel beam from a synchrotron. The analyser therefore requires the combination of a CDW system and the very large-acceptance angle collimating optics.

Early experiments had demonstrated the existence of angular dispersion in asymmetric Bragg diffraction^24^. Various components of the UHRIX spectrometer have been tested separately during the last few years in a series of experiments. The initial CDW design had been tested^24^,^25^,^26^,^27^ and improved by implementing doubly-dispersive CDFDW^22^,^28^,^29^ and CDDW optics^23^. A Montel-type two-dimensional (2D) mirror system was designed and tested to work as the large-acceptance angle collimating optics^30^.

Although various components of the spectrometer have been developed, refined and tested, they have never before been combined in a fully functional spectrometer. The challenge and the final proof of the concept is not just in a proper functioning of each individually intricate element, but in a precise coupling of all optic elements. The flat-crystal CDDW monochromator and curved focusing mirror (Fig. 3) have to be properly designed and coupled to produce not only high spectral flux but also about 10-μm small vertical focal spot size on the sample despite the strong angular dispersion in the CDDW monochromator^29^. The small size of the secondary source is essential for the collimating mirror to collect photons in the large solid angle Δϕ_*x*_ × Δϕ_*y*_≲10 × 10 mrad^2^ and to produce sufficiently parallel 100 μrad beam for proper coupling with the CDFDW analyser (Fig. 3). Only flawless coupling of all elements may result in the highest resolution and efficiency of the spectrometer.

### Spectrometer momentum transfer and spectral resolution functions

The experiments were performed at the undulator beamline 30ID at the Advanced Photon Source. The UHRIX instrument is shown schematically in Fig. 3 and presented in detail in Methods. Once the CDDW monochromator and the Kirkpatrick-Baez (KB) focusing mirrors had brought a large flux ≃2.4 × 10^9^ ph s^−1^ of ultra-monochromatic Δ*E*_i_=0.25 meV X-rays to a ≃18(V) × 45(H)-μm^2^ spot on the sample, it remained to test the new analyser system. Measurements of the momentum and spectral resolution functions, and of IXS spectra, are the ultimate proof of the new concept.

**Figure 4:**
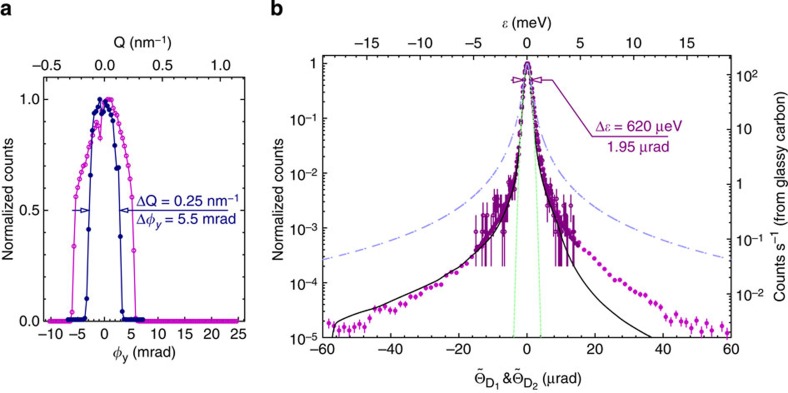
UHRIX resolution functions. (**a**) Momentum transfer resolution Δ*Q* of the UHRIX instrument measured by the analyser system rotated about *y* axis with angle *ϕ*_*y*_. Open circles: Δ*Q*=0.49 nm^−1^, corresponding to the collimating mirror and CDFDW optics being fully open. Solid circles: Δ*Q*=0.25 nm^−1^, with a horizontal aperture installed after the collimating Montel optics, to reduce the solid angle of the analyser system acceptance. (**b**) UHRIX spectral resolution functions measured by scanning the CDDW monochromator energy. Light purple solid circles: the analyser system is at *ϕ*_*y*_=0 intercepting the direct focused monochromatic X-ray beam. Dark purple open circles: the analyser system is at ϕ_*y*_=21.6 mrad (*Q*=1 nm^−1^) intercepting X-rays scattered from a strong elastic scatterer, glassy carbon. The black solid line is the convolution of the theoretical spectral functions of the CDDW and CDFDW optics calculated using multi-crystal dynamical theory of X-ray diffraction. Other functions with the same FWHM are shown for comparison: dashed green line—Gaussian; blue dash-dotted line—Lorentzian.

**Figure 5:**
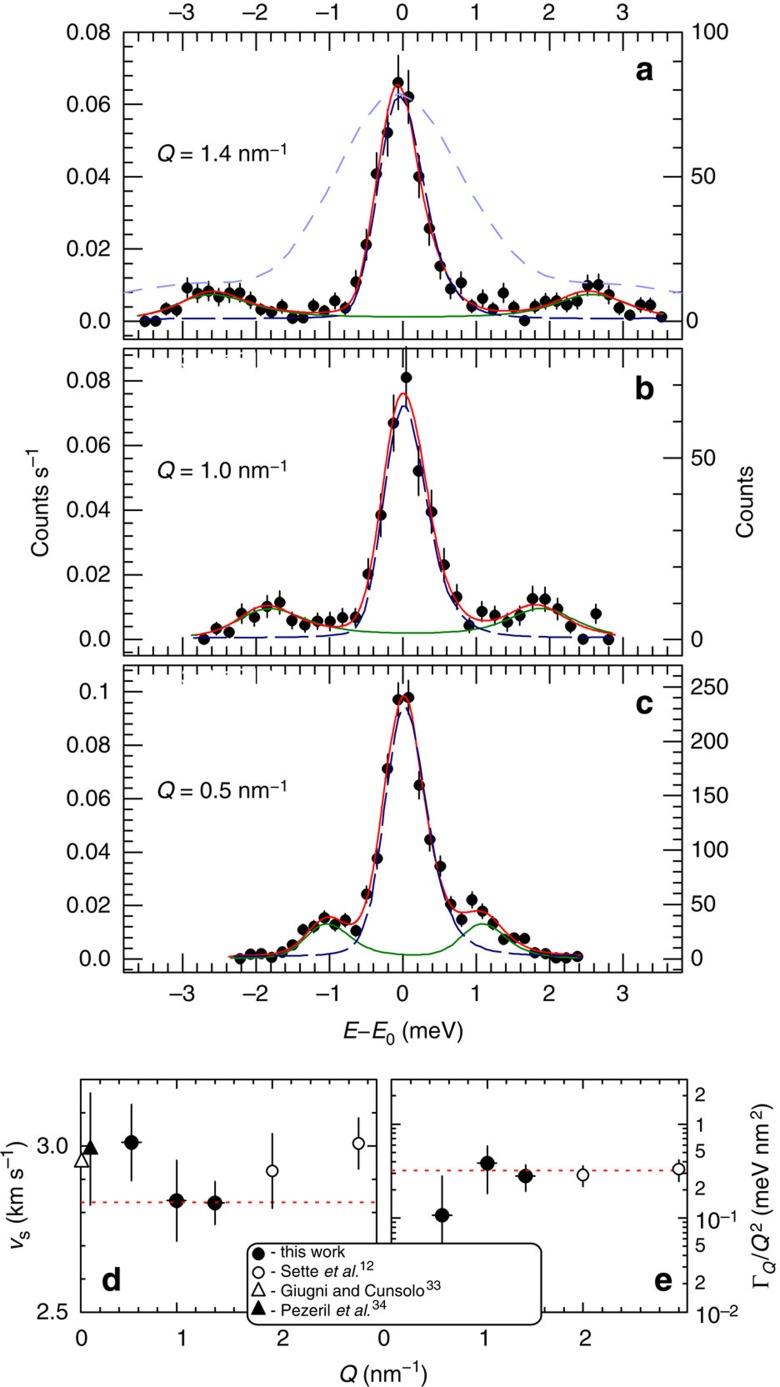
Glycerol IXS spectra, sound velocity and attenuation. IXS spectra of glycerol liquid at 298 K measured at different momentum transfer *Q*=0.5 nm^−1^ (**a**), *Q*=1 nm^−1^ (**b**) and *Q*=1.4 nm^−1^ (**c**), respectively. Dashed dark-blue lines are UHRIX resolution functions. Solid green lines are damped harmonic oscillator (DHO) functions convoluted with the resolution function. Solid red lines are a sum of the two, fitted to the experimental spectra. The dashed light-blue line in **c** is an IXS spectrum of glycerol measured with a conventional IXS spectrometer at *Q*=1.5 nm^−1^ (ref. 31). The apparent sound velocity 
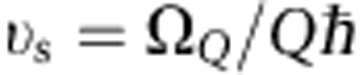
 (**d**) and the reduced broadening Г_*Q*_/*Q*^2^ (**e**) of the acoustic-like modes, obtained from the least-squares fit of the experimental data to the DHO model, are shown with black circles and ±1*σ* error bars.

UHRIX momentum transfer resolution measurements are presented in Fig. 4a. The spectrometer was tested in two modes, first by accepting the entire beam onto the analyser, which produced the energy resolution shown in Fig. 4b, and momentum resolution Δ*Q*=0.49 nm^−1^. A 1-mm horizontal slit was then placed before the analyser to reduce the momentum resolution to Δ*Q*=0.25 nm^−1^ and produce the IXS spectra shown in Fig. 5.

UHRIX spectral resolution functions are shown in Fig. 4b, in which only the CDDW monochromator energy is scanned. We show that the spectral line shape measured from the direct beam, and that from the beam diverging from a glassy carbon amorphous sample, are identical. Both results show a 620-μeV spectral bandwidth that was never before achieved with an IXS spectrometer and is in good agreement with the predictions of dynamical diffraction theory. In addition to the factor of three improvements in bandwidth, the UHRIX resolution function exhibits vastly improved contrast. It has an almost Gaussian shape over two orders of magnitude in intensity and is an order of magnitude steeper than the Lorentzian shape that is typical for existing IXS spectrometers. The improved momentum resolution, better spectral resolution and higher contrast are game changers for IXS spectroscopy.

### Spectrometer applied to studies of dynamics in glycerol

We have applied our new spectrometer to a study of glycerol, a prototypical glass-forming liquid. Figure 5 shows IXS spectra measured with momentum transfers of *Q*=1.4, 1.0 and 0.5 nm^−1^. All the spectra clearly reveal Stokes and anti-Stokes inelastic lines, interpreted as longitudinal acoustic-like modes in glycerol, well resolved from the central elastic line, even at the lowest *Q*. For comparison, we show measurements in glycerol^31^ with a state-of-the-art conventional IXS spectrometer at *Q*=1.5 nm^−1^—the smallest accessible for such spectrometers. Unlike the UHRIX measurements, the inelastic features appear unresolved as shoulders on the elastic line tail. UHRIX can resolve phonon lines even at *Q*=0.5 nm^−1^, due to its high resolution and high contrast capabilities, where conventional spectrometers fail.

The IXS data are modelled by the normalized dynamical structure factor


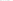


the variable measured in IXS experiments^13^, presented here as a sum of the delta function for the elastic component and the damped harmonic oscillator for the inelastic component^32^. Convolution of *S*(*Q*,*E*)/*S*(*Q*) with the instrumental function is used to compare with the experimental results and derive the model parameters *f*_Q_, *Г*_Q_ and Ω_Q_. See Fig. 5, which also shows the apparent sound velocity 
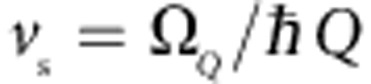
 and the reduced broadening *Г*_Q_/*Q*^2^ of the acoustic-like modes in glycerol, obtained from the least-squares fit of the experimental data.

Speed of sound *v*_s_ in Fig. 5d is flat at *Q*=1 and 1.4 nm^−1^, and is smaller than the values measured at larger *Q* in earlier IXS studies in glycerol liquid^12^, shown by open circles. At the smallest measured *Q*=0.5 nm^−1^, it increases to the level determined by low-frequency techniques^33^,^34^, as shown with triangles. At this point the reduced width *Г*_Q_/*Q*^2^ in Fig. 5e drops down from the constant level measured at larger *Q* in the present and earlier IXS studies. This indicates a breakdown of the *Г*_Q_∝*Q*^2^ dependence typical for liquid and glass dynamics in the THZ frequency range discovered in early IXS studies^12^. The low-frequency measurements in glycerol liquid revealed a linear dependence in *Q* and much smaller attenuation^34^. Therefore, breakdown of the *Q*^2^ dependence in the intermediate frequency and *Q* range is a plausible scenario. Breakdown of the *Q*^2^ and cross-over to a faster *Q*^4^ dependence have been reported in glasses^35^, and in particular very clearly in glycerol glass at *T*=150 K^16^. Our observation of the simultaneous increase of *ν*_s_ and breakdown of *Q*^2^ dependence is very similar to the observations reported for glycerol glass^16^. The results of the present studies in liquid glycerol indicate that such behaviour cannot be taken as a unique identifier of a glass. Our results indicate that similar behaviour occurs in glass-forming liquids at longer time–length scales, compared with those typical for glasses, precisely in the regime that has become accessible with the novel IXS spectrometer demonstrated in this paper.

## Discussion

A conceptually new IXS spectrometer was demonstrated, which opens up hitherto inaccessible region of the time- and length-scale landscape of collective excitations in condensed matter. Novel X-ray crystal optics combined with special collimating optics result in unmatched performance. The spectrometer features a spectral resolution function with Gaussian-like steep tails over two orders of magnitude in intensity and sub-meV (≃620 μeV) bandwidth. The new capabilities are demonstrated by studies of dynamics in glass-forming glycerol liquid, with a momentum transfer resolution of 0.25 nm^−1^. The moderate working photon energy of 9.1 keV makes this spectrometer practical for most X-ray synchrotron and X-ray free-electron laser (XFEL) facilities.

The optical principles demonstrated here can be further developed towards achieving an even greater spectroscopic performance. Complemented with additional focusing optics and a position sensitive detector, the analyser system can be transformed into a spectrograph imaging single-shot spectra with sub-0.1-meV resolution, as has recently been demonstrated in a proof-of-principle experiment^28^. A 2D detector would allow single-shot imaging of IXS spectra at different *Q*-values with sub-0.1-nm^−1^ resolution. The X-ray spectrometer concept demonstrated here, which combines flat crystal angular dispersive optics with focusing and collimating optics is a new and exciting paradigm, which is highly attractive for a wide range of applications in non-resonant and resonant IXS.

### Spectrometer details

The experiments were performed at the undulator beamline 30ID at the Advanced Photon Source^36^. The layout of the UHRIX instrument is shown schematically in Fig. 3. The undulator X-ray source and the water-cooled double-crystal diamond pre-monochromator were tuned to a nominal X-ray photon energy *E*_i_=9.1315, keV of the UHRIX instrument. Final monochromatization of X-rays incident on the sample was achieved with a hybrid diamond-silicon CDDW monochromator^23^, a modification of the CDFDW analyser^22^. An asymmetrically cut 200-μm-thick silicon (220) crystal plate used as the CFW element in the CDFDW optics was replaced with a 100-μm-thick asymmetrically cut diamond (133) plate. Unlike silicon, the thin diamond plate is practically transparent for the 9-keV photons and therefore functions as a CW element in the CDDW optics. The substitution with the diamond CW element resulted in a very small energy bandwidth Δ*E*_i_=0.25 meV, a threefold increase in the aperture of the accepted beam, a reduction in the cumulative angular dispersion rate of X-rays emanating from the monochromator for better focusing on a sample, a sufficient angular acceptance matching the angular divergence of an undulator source (≃10 μrad) and a very high spectral efficiency of 65% due to low X-ray absorption in the thin diamond crystal. The CDDW monochromator energy *E*_i_ can be scanned by simultaneous rotation of the two D-crystals, as indicated by rotation angles 
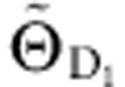
 and 
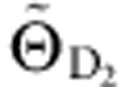
 in Fig. 3. A downstream KB mirror system focused X-rays on the sample to a 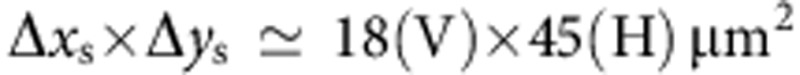 spot. A flux of 2.4 × 10^9^ photons/s was delivered to the sample.

### Analyser system

The most critical but unexplored component before our experiment was the analyser system comprising a collimating optics and the CDFDW crystal analyser^22^. A Montel-type 2D mirror system was used as the collimating optics^30^. As predicted, the Montel optics collected X-rays in a solid angle Δ*ϕ*_*x*_ × Δ*ϕ*_*y*_≲10 × 10 mrad^2^ and collimated them to a beam with a much smaller divergence Δ*ψ*_*x*_ × Δ*ψ*_*y*_≲90(V) × 230(H)  μrad^2^. The vertical angular spread Δ*ψ*_*x*_ was well matched to the 105 μrad angular acceptance of the CDFDW analyser^22^ and ensured its high efficiency. Keeping the horizontal angular spread Δ*ψ*_*y*_ small was also important, to avoid broadening of the CDFDW spectral bandwidth. For the backscattering CDW-type optics, the broadening was estimated as 
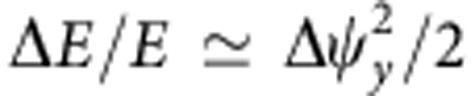
 (ref. 21), that is, Δ*E*≃0.24 meV in our case, which was within acceptable limits. The measured values of the angular spread Δ*ψ*_*y*_ × Δ*ψ*_*x*_ correspond to the angular size (Δ*y*_s_/*f*_M_) × (Δ*x*_s_/*f*_M_) of the secondary source (sample) seen from the centre of the Montel optics at a focal distance of *f*_M_=0.2 m, emphasizing the importance of the small secondary source size for proper functioning of the analyser system.

### Collimating optics

The collimating Montel optics, manufactured by Incoatec GmbH, consisted of two parabolically shaped, 120-mm-long surfaces coated with laterally graded 100 W–C bilayers (Göbel mirrors), arranged perpendicularly and side-by-side^30^. An almost 50% reflectivity of the Montel optics (≃70% from each surface) was measured with the analyser system set at *ϕ*_*y*_=0 looking into the direct focused beam. The angular spread of X-rays from the Montel optics was measured with a Si(220) channel-cut crystal installed downstream temporarily for this measurements. In this case, X-rays were scattered from a 1-mm-thick amorphous carbon plate sample, and the analyser system set to *ϕ*_*y*_≃22 mrad (*Q*=1 nm^−1^).

### Momentum transfer resolution

Momentum transfer resolution of the instrument was limited by two factors: the angular spread of the incident beam and the angular acceptance of the analyser system. The incident beam divergence due to focusing onto the sample is ≲0.4(V) × 0.8(H)  mrad^2^ and therefore can add only a small broadening of ≲0.016(V) × 0.037(H) nm^−2^ to the momentum transfer resolution. Although the vertical and horizontal angular acceptance of the Montel optics were the same, that of the analyser system in the vertical plane was presently limited to Δ*ϕ*_*x*_=0.75 mrad by the 90-mm-length of the D-crystals in the CDFDW optics used in this experiments. The angular acceptance of the analyser system in the horizontal plane made the main contribution to the momentum transfer resolution function.

With no sample installed, the focused beam propagating in the forward direction was used to align the collimating Montel and the downstream CDFDW optics, and to measure the UHRIX resolution functions. Rotation of the analyser system in the horizontal plane by an angle *ϕ*_*y*_ about the vertical axis passing through the sample provided access to photons scattered with a momentum transfer *Q*=2*k*_i_ sin(*ϕ*_*y*_/2).

### Sample

The liquid glycerol sample (*T*=298 K) was contained between two 12-μm-thick Kapton windows with a separation of 1.5 mm, matched to the X-ray absorption length. To avoid possible radiation damage, the liquid was pumped through the container.

## Author contributions

Y.S. planned and organized the work on UHRIX development, performed experiments, analysed the data and wrote the manuscript. S.S. built and tested UHRIX spectrometer major components, including CDDW and CDFDW optics, and performed experiments. D.S. made mechanical design of the spectrometer components. S.P.C. organized the work on collimating optics, performed experiments and co-wrote the manuscript. K.M. and J.S. developed collimating optics and performed experiments. M.T. performed experiments and evaluated the data.

## Additional information

**How to cite this article**: Shvyd’ko, Y. *et al.* High-contrast sub-millivolt inelastic X-ray scattering for nano- and mesoscale science. *Nat. Commun.* 5:4219 doi: 10.1038/ncomms5219 (2014).

## Figures and Tables

**Figure 1:**
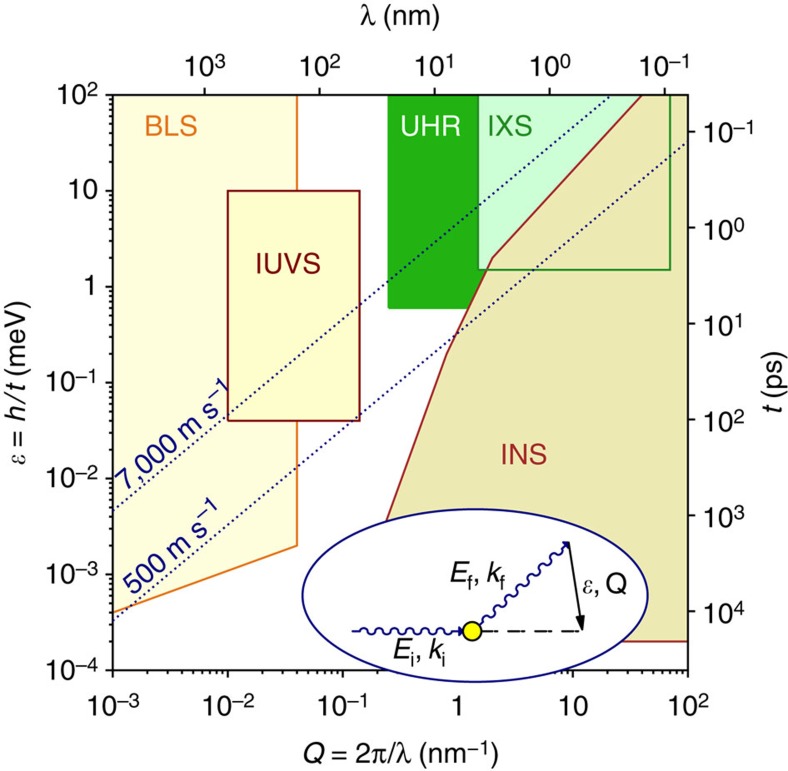
Accessibility of time-length space by different techniques. Time–length 
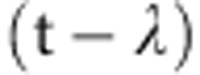
 and relevant energy–momentum (*ε*–*Q*) space of excitations in condensed matter. Coloured regions show how it is accessed by different inelastic scattering probes: inelastic neutron scattering (INS), inelastic x-ray scattering (IXS), inelastic UV scattering (IUVS) and Brillouin scattering (BLS). The UHRIX spectrometer presented in the paper enters the previously inaccessible region marked in dark green. The energy *ε*=*E*_f_–*E*_i_ and the momentum *Q*=*k*_f_−*k*_i_ transfers from initial to final photon/neutron states are measured in inelastic scattering experiments, as shown schematically in the oval inset.

## References

[b1] Anderson P. Through the glass lightly. Science 267, 1615 (1995). 17808155 10.1126/science.267.5204.1615-e

[b2] Angell C. A. Formation of glasses from liquids and biopolymers. Science 267, 1924–1935 (1995). 17770101 10.1126/science.267.5206.1924

[b3] Fox K. C. Putting proteins under glass. Science 267, 1922–1923 (1995). 7701317 10.1126/science.7701317

[b4] Debenedetti P. G. & Stillinger F. H. Supercooled liquids and the glass transition. Nature 410, 259–267 (2001). 11258381 10.1038/35065704

[b5] Donth E. The Glass Transition-Relaxation Dynamics in Liquids and Disordered Materials Vol. 48 of Material SciencesSpringer: Berlin, Heidelberg, New York, (2001).

[b6] Scopigno T., Ruocco G., Sette F. & Monaco G. Is the fragility of a liquid embedded in the properties of its glass? Science 302, 849–852 (2003). 14593174 10.1126/science.1089446

[b7] Novikov V. & Sokolov A. Poisson’s ratio and fragility of glass-forming liquids. Nature 431, 961–963 (2004). 15496916 10.1038/nature02947

[b8] Brockhouse B. N. & Stewart A. T. Normal modes of aluminum by neutron spectrometry. Rev. Mod. Phys. 30, 236–249 (1958).

[b9] Burkel E., Dorner B. & Peisl J. Observation of inelastic x-ray scattering from phonons. Europhys. Lett. 3, 957–961 (1987).

[b10] Sette F. *et al.* Collective dynamics in water by high energy resolution inelastic x-ray scattering. Phys. Rev. Lett. 75, 850–853 (1995). 10060134 10.1103/PhysRevLett.75.850

[b11] Masciovecchio C. *et al.* A perfect crystal x-ray analyzer with 1.5 meV energy resolution. Nucl. Instrum. Methods Phys. Res. B 117, 339–340 (1996).

[b12] Sette F., Krisch M. H., Masciovecchio C., Ruocco G. & Monaco G. Dynamics of glasses and glass-forming liquids studied by inelastic x-ray scattering. Science 280, 1550–1555 (1998).

[b13] Burkel E. Phonon spectroscopy by inelastic x-ray scattering. Rep. Prog. Phys. 63, 171–232 (2000).

[b14] Sinn H. *et al.* Microscopic dynamics of liquid aluminum oxide. Science 299, 2047–2049 (2003). 12663922 10.1126/science.1080950

[b15] Scopigno T., Ruocco G. & Sette F. Microscopic dynamics in liquid metals: the experimental point of view. Rev. Mod. Phys. 77, 881–933 (2005).

[b16] Monaco G. & Giordano V. M. Breakdown of the Debye approximation for the acoustic modes with nanometric wavelengths in glasses. PNAS 106, 3659–3663 (2009). 19240211 10.1073/pnas.0808965106 PMC2656136

[b17] Baldi G., Giordano V. M., Monaco G. & Ruta B. Sound attenuation at terahertz frequencies and the boson peak of vitreous silica. Phys. Rev. Lett. 104, 195501 (2010). 20866974 10.1103/PhysRevLett.104.195501

[b18] Baron A. Q. R. *et al.* Early commissioning of the SPring-8 beamline for high resolution inelastic x-ray scattering. Nucl. Instrum. Methods Phys. Res. A 467-468, 627–630 (2001).

[b19] Sinn H. *et al.* An inelastic x-ray spectrometer with 2.2 meV energy resolution. Nucl. Instrum. Methods Phys. Res. A 467-468, 1545–1548 (2001).

[b20] Said A. H., Sinn H. & Divan R. New developments in fabrication of high-energy-resolution analyzers for inelastic X-ray spectroscopy. J. Synchrotron Radiat. 18, 492–496 (2011). 21525659 10.1107/S0909049511001828 PMC3268696

[b21] Shvyd'ko Y. X-Ray Optics – High-Energy-Resolution Applications Vol. 98 of Optical SciencesSpringer: Berlin, Heidelberg, New York, (2004).

[b22] Shvyd'ko Y. u., Stoupin S., Shu D. & Khachatryan R. Using angular dispersion and anomalous transmission to shape ultramonochromatic x rays. Phys. Rev. A 84, 053823 (2011).

[b23] Stoupin S. *et al.* Hybrid diamond-silicon angular-dispersive x-ray monochromator with 0.25-mev energy bandwidth and high spectral efficiency. Opt. Express 21, 30932–30946 (2013). 24514666 10.1364/OE.21.030932

[b24] Shvyd'ko Y. V. *et al.* X-ray Bragg diffraction in asymmetric backscattering geometry. Phys. Rev. Lett. 97, 235502 (2006). 17280212 10.1103/PhysRevLett.97.235502

[b25] Shvyd'ko Y. u. V. *et al.* Progress in the development of new optics for very high resolution inelastic x-ray scattering spectroscopy. AIP Conf. Proc. 879, 737–745 (2007).

[b26] Stetsko Y. P. *et al.* Multiple-wave diffraction in high energy resolution back-reflecting x-ray optics. Phys. Rev. Lett. 107, 155503 (2011). 22107300 10.1103/PhysRevLett.107.155503

[b27] Cai Y. Q. *et al.* The ultrahigh resolution ixs beamline of NSLS-II: Recent advances and scientific opportunities. J. Phys. Conf. Ser. 425, 202001 (2013).

[b28] Shvyd'ko Y., Stoupin S., Mundboth K. & Kim J. Hard-x-ray spectrographs with resolution beyond 100 μev. Phys. Rev. A 87, 043835 (2013).

[b29] Shu D. *et al.* Precision mechanical design of an ultrahigh-resolution inelastic x-ray scattering spectrometer system with cdfdw optics at the aps. J. Phys. Confer. Ser. 425, 052031 (2013).

[b30] Mundboth K. *et al.* Tests and characterization of a laterally graded multilayer Montel mirror. J. Synchrotron Radiat. 21, 16–23 (2014). 24365912 10.1107/S1600577513024077

[b31] Cunsolo A., Leu B. M., Said A. H. & Cai Y. Q. Structural and microscopic relaxations in glycerol: an inelastic x-ray scattering study. J. Chem. Phys. 134, 184502 (2011). 21568516 10.1063/1.3587104

[b32] Monaco G., Cunsolo A., Ruocco G. & Sette F. Viscoelastic behavior of water in the terahertz-frequency range: an inelastic x-ray scattering study. Phys. Rev. E 60, 5505–5521 (1999). 10.1103/physreve.60.5505 11970425

[b33] Giugni A. & Cunsolo A. Structural relaxation in the dynamics of glycerol: a joint visible, uv and x-ray inelastic scattering study. J. Phys. Condensed Matter 18, 889–902 (2006).

[b34] Pezeril T., Klieber C., Andrieu S. & Nelson K. A. Optical generation of gigahertz-frequency shear acoustic waves in liquid glycerol. Phys. Rev. Lett. 102, 107402 (2009). 19392158 10.1103/PhysRevLett.102.107402

[b35] Rufflé B., Guimbretière G., Courtens E., Vacher R. & Monaco G. Glass-specific behavior in the damping of acousticlike vibrations. Phys. Rev. Lett. 96, 045502 (2006). 16486840 10.1103/PhysRevLett.96.045502

[b36] Shvyd'ko Y. *et al.* MERIX - next generation medium energy resolution inelastic x-ray scattering instrument at the APS. J. Electron Spectrosc. Relat. Phenom. 188, 140–149 (2013).

